# Comparison of everolimus-eluting and biolimus-eluting coronary stents with everolimus-eluting bioresorbable scaffold: study protocol of the randomized controlled EVERBIO II trial

**DOI:** 10.1186/1745-6215-15-9

**Published:** 2014-01-07

**Authors:** Diego Arroyo, Mario Togni, Serban Puricel, Baeriswyl Gerard, Lehmann Sonja, Noé Corpataux, Hélène Villeneuve, Estelle Boute, Jean-Christophe Stauffer, Jean-Jacques Goy, Stéphane Cook

**Affiliations:** 1 Department of Cardiology, University & Hospital, Fribourg, Switzerland

**Keywords:** Biolimus-eluting stent, Bioresorbable vascular scaffold (BVS), Coronary artery disease, Drug-eluting stent, Everolimus-eluting stent, Late lumen loss, Percutaneous coronary intervention

## Abstract

**Background:**

Second-generation everolimus-eluting stents (EES) and third generation biolimus-eluting stents (BES) have been shown to be superior to first-generation paclitaxel-eluting stents (PES) and second-generation sirolimus-eluting stents (SES). However, neointimal proliferation and very late stent thrombosis is still an unresolved issue of drug-eluting stent (DES) implantation overall. The Absorb™ (Abbott Vascular, Abbott Park, IL, USA) is the first CE approved DES with a bioresorbable vascular scaffold (BVS) thought to reduce long-term complication rates. The EVERBIO II trial was set up to compare the BVS safety and efficacy with both EES and BES in all patients viable for inclusion.

**Methods/Design:**

The EVERBIO II trial is a single-center, assessor-blinded, randomized trial. The study population consists of all patients aged ≥18 years old undergoing percutaneous coronary intervention. Exclusion criterion is where the lesion cannot be treated with BVS (reference vessel diameter >4.0 mm). A total of 240 patients will be enrolled and randomly assigned into 3 groups of 80 with either BVS, EES or BES implantation. All patients will undergo a follow-up angiography study at 9 months. Clinical follow-up for up to 5 years will be conducted by telephone. The primary endpoint is in-segment late lumen loss at 9 months measured by quantitative coronary angiography. Secondary endpoints are patient-oriented major adverse cardiac event (MACE) (death, myocardial infarction and target-vessel revascularization), device-oriented MACE (cardiac death, myocardial infarction and target-lesion revascularization), stent thrombosis according to ARC and binary restenosis at follow-up 12 months angiography.

**Discussion:**

EVERBIO II is an independent, randomized study, aiming to compare the clinical efficacy, angiographic outcomes and safety of BVS, EES and BES in all comer patients.

**Trial registration:**

The trial listed in clinicaltrials.gov as NCT01711931.

## Background

Drug-eluting stents (DES) have considerably reduced neointimal hyperplasia and significantly decreased the risk of restenosis compared to bare metal stents (BMS) [1]. However, the persistent polymer within the vessel lumen has been held responsible for ongoing endothelial inflammation, incomplete endothelialization and subsequent atherosclerosis, all leading to late complications such as restenosis and stent thrombosis [2].

Everolimus-eluting stents (EES) and biolimus-eluting stents (BES) are more recent DES that have been widely investigated. The EES is at present the most frequently used DES in the USA and in Europe [3]. It was demonstrated as non-inferior to sirolimus-eluting stents (SES) in the ISAR-TEST IV and the EXCELLENT trial and superior to paclitaxel eluting stents (PES) in two large randomized studies (Spirit-IV and COMPARE) [4-7]. One propensity score matched registry (LESSON-1) showed a trend towards a lower risk of death, myocardial infarction (MI) and target-vessel revascularization (TVR) as compared to SES over a 3-year follow-up [8].

BES differs from EES in that it possesses an abluminal polymer coating that is completely converted to lactic acid in 6 months and, via the Krebs cycle, to carbon dioxide and water in 6 to 9 months. Two large clinical trials (NOBORI and LEADERS) proved its non-inferiority to PES and SES at 9 months and 4 years, respectively [9,10]. The propensity score matched EVERBIO trial showed similar risk of death, MI and TVR in BES compared to EES during a 2-year follow-up [11]. Recently, the randomized COMPARE II trial enrolling 2,707 patients, confirmed the BES safety but no differences were observed in clinical endpoints at 2-year follow-up when compared to EES [12]. The ongoing Global LEADERS trial with an expected inclusion of >10,000 patients will provide further information on BES. The lactic acidification of the media from the polymer could impact vascular healing at the stent vicinity and promote a deleterious inflammatory reaction [13].

The unresolved problem of neointimal proliferation and very late stent thrombosis from lingering polymers and vascular scaffolds, led to the development of new-generation completely resorbable stents. The Absorb™ (Abbott Vascular, Abbott Park, IL, USA) is the first CE approved DES with a bioresorbable vascular scaffold (BVS). It uses a poly-l-lactide polymer that is absorbed after approximately 2 years via the Krebs cycle. A prospective, open-label, 2-stage study called ABSORB including 131 patients was conducted in Europe and New Zealand and led to its approval by the EU in January 2011 for the treatment of coronary artery lesions [14]. The major adverse cardiac event (MACE) rate was reported as 6.8% at 2-years follow-up and late lumen loss (LLL) 0.27 mm at 12 months [15,16]. Its non-inferiority to the Xience Prime (Abbott Vascular), an EES, is currently under investigation in the international ABSORB EXTEND trial that plans to enroll 1,000 patients (NCT01023789). To date, there is no data on BVS compared to EES or BES, and independent trials are eagerly awaited. We seek to compare the efficacy and safety profiles of these three different types and generations of DES.

### Study objectives and hypothesis

The purpose of the EVERBIO II trial is to evaluate the efficacy, angiographic outcome and safety of three different stents in *de novo* coronary artery lesions: the BVS Absorb™ (Abbott Vascular), the EES Promus Element™ (Boston Scientific, Natick, MA, USA) and the BES Biomatrix Flex™ (Biosensors International Ltd., Morges, Switzerland). The null hypothesis to be rejected is that all three stents are of equal efficacy. We believe there will be a significant difference with regard to LLL at 9 months and a clinical endpoint of death, MI and TVR at 12 months between EES and BES and BVS stents.

## Methods/Design

### Study design and overview

**Figure 1:**
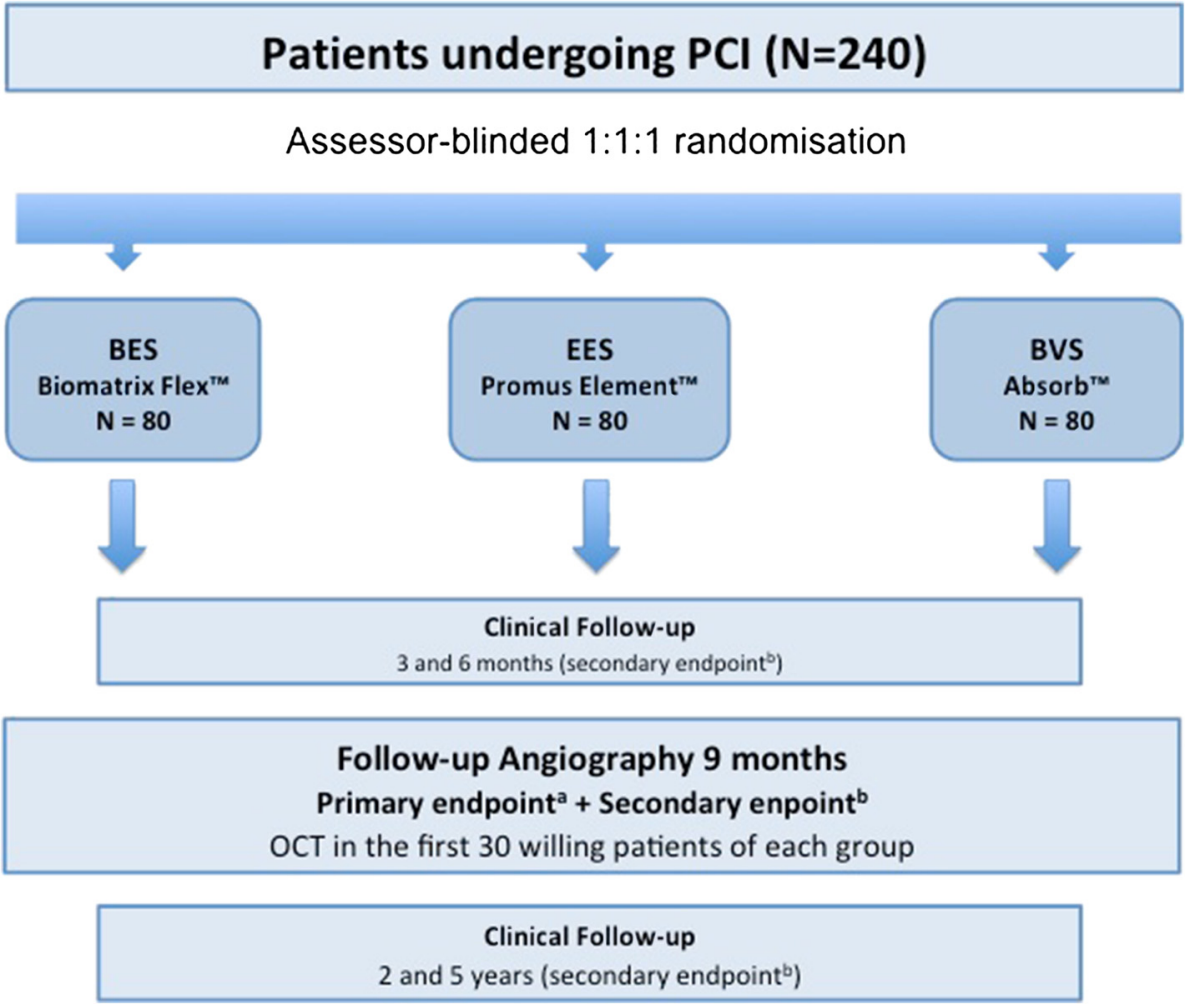
**Study algorithm.** BES: biolimus-eluting stent, BVS: biovascular scaffold, EES: everolimus-eluting stent, OCT: optical coherence tomography, PCI: percutaneous coronary intervention. ^a^Primary endpoint: late lumen loss at 9-month angiography study. ^b^Secondary endpoints: patient oriented (death, myocardial infarction, target vessel revascularization), device oriented (cardiac death, myocardial infarction, target-lesion revascularization), stent thrombosis, binary restenosis at 9-month angiography study.

This is a single center, assessor-blinded, randomized study comparing three different stents in *de novo* coronary lesions: the Absorb™, the Promus Element™ and the Biomatrix Flex™. The protocol of the trial has been registered online (NCT01711931) at http://www.clinicaltrials.gov. Figure 1 briefly summarizes the main study steps and Table 1 the longitudinal follow-up. The organization and scientific conduct is supervised by a Steering Committee. A Data and Safety Monitoring Board is responsible for safety and ethical aspects. A Clinical Events Adjudication Committee (CEAC) including interventional and non-interventional cardiologists review and adjudicate all reported events and endpoints and perform computation and angiographic measurements. All members of the CEAC are blinded to the primary results of the trial. The study complies with the Declaration of Helsinki and was approved by the local ethics committee of Fribourg University and Hospital (Switzerland, 043/12-CER-FR).

**Table 1:** Timetable of prospective investigations

**Investigation**	**Baseline**	**Post procedure**	**3 months**	**6 months**	**9 months**	**12 months**	**2 years**	**5 years**
Medical/clinical history	x	x	x	x	x	x	x	x
Physical examination	x	x			x			
12-lead ECG^a^	x	x			x			
Coronary angiogram^b^	x				x			
Procedural complications		x			x			
Laboratory survey^c^	x				x			
CK, CK-MB, troponin^d^		x			x			
Medications	x	x	x	x	x	x	x	x
Primary outcome					x			
Secondary outcome			x	x	x	x	x	x

^a^Any ECG may be obtained when clinically relevant.

^b^Unscheduled angiograms after 6 months will be considered 9-month follow-up angiograms.

^c^Usual laboratory survey include, complete blood count, electrolytes, fasting glucose level, INR, prothrombin time, partial thromboplastin time, creatinine, blood urea nitrogen, pregnancy test if applicable.

^d^All patients will benefit from cardiac enzyme measurement 4 h post procedure, enzymes may be followed every 8 h if clinically relevant and at the discretion of the operator.

*CK* creatine kinase, *ECG* electrocardiogram.

### Study endpoints

The primary endpoint is LLL at 9 months as assessed by quantitative coronary angiography. The secondary endpoints are divided in angiographic and clinical findings. We will assess angiographic success, device success and binary restenosis at 9 months. Clinical endpoints include a patient-oriented MACE (composite of death, MI and TVR) a device-oriented MACE (composite of cardiac death, MI and target lesion revascularization (TLR)) and stent thrombosis (ST) at 6 months, and 1, 2 and 5 years.

### Patient selection criteria

All patients aged ≥18 years undergoing coronary angiography at the University & Hospital Fribourg (Switzerland) for suspected coronary artery disease on functional cardiac testing, stable angina or acute coronary syndrome (unstable angina, non-ST segment MI, ST-elevated MI) are eligible. Patients must be apt and willing to provide written informed consent and participate in follow-up. Patients with a known or presumed hypersensitivity to heparin, antiplatelet drugs and hypersensitivity to contrast dye not controllable with standard premedication will be excluded. Patients are recruited on the day of their angiography by one of the investigators if all inclusion criteria are met and no exclusion criteria apply. Written informed consent will be obtained as required by the local institutional ethics committee in accordance with the Declaration of Helsinki.

### Treatment assignment

Patient randomization will be performed after lesion preparation. Exclusion criterion is when the lesion cannot be treated with BVS (reference vessel diameter >4.0 mm). Randomization will be completed via computer-generated numbers. Allocation concealment will be kept in sealed envelopes containing a non-transparent pleated color sheet in which the etiquette for intervention is embedded. The investigator is responsible for enrolment and a study nurse will assign participants to either stent implantation. Only the outcome assessors and data analysts are blinded to the intervention.

### Index percutaneous coronary intervention

Procedures will be performed via the femoral or radial artery with a 5-6 French (F) guiding catheter. Standard interventional techniques will be used and performed according to practice guidelines. Preprocedural antithrombotic regimen is systematically achieved with aspirin (500 mg intravenous bolus for those not under treatment and 100 mg for those already under aspirin and then 100 mg/day for all) and unfractionated heparin (70 UI/kg), whereas glycoprotein IIb/IIIa inhibitors are used per operator discretion. All patients will receive either a minimum 600 mg loading dose of clopidogrel, 180 mg of ticagrelor, or 60 mg prasugrel before or immediately after the procedure. Lifelong ≥100 mg daily aspirin and either 75 mg daily clopidogrel, or 90 mg twice-daily ticagrelor or 10 mg prasugrel for a minimum of 6 months will be prescribed. Other medications will be prescribed as per standard of care. All patients will be monitored between 4 to 12 h in an intermediate care unit and will undergo baseline and 3 to 6 h cardiac biomarker measurements. A standard 12-lead electrocardiogram (ECG) is recorded immediately after the procedure and with each biomarker measurement.

### Follow-up coronary angiography

Effectiveness will be measured at the 9-month follow-up angiography in all patients. Quantitative measures will be performed within the stent and the 5 mm edge region. In-segment LLL will be defined as the difference between the minimum lumen diameter post procedure and at 9 months. Binary restenosis will be defined as >50% diameter stenosis. LLL is computed through quantitative coronary angiography with the use of automated edge-detection system (CAAS II, Pie Medical Imaging, Maastricht, The Netherlands). Any unscheduled angiogram after 6 months will be considered as 9-month follow-up angiogram.

Optical coherence tomography (OCT) is conducted in the first 30 patients of each group willing to undergo the aforementioned procedure. Percentage of malapposed struts, frequency of abnormal intrastent tissue, frequency of peristrut low intensity area and percent net volume obstruction will be assessed with the use of automated edge-detection system (Illumien-optis ORW, St. Jude Medical, St. Paul, MN, USA).

### Clinical follow-up

Patients will be followed clinically by clinic visits or by telephone interview at 3, 6, 9 and 12 months, and 2 and 5 years. No blinding will be present during the collection of outcome data. If the patient is not accessible, data can be retrieved from the referring physician or the hospital electronic database.

### Statistical analyses

We plan to enroll 80 patients in each group. Comparison between EES and BES with BVS will necessitate a total of 240 patients in order to achieve a power of 90% to prove a difference of 0.2 mm LLL at 9 months (BES and EES = 0.3 mm vs BVS 0.5 mm; SD 0.5 mm). This allows for a dropout rate of 20% of the sample in which case the study would still yield a power of 83%. Sample size calculations were performed using Sample Power 3 (SPSS Inc, Chicago, IL, USA) at a two-tailed significance level of α = 0.05.

Continuous variables are presented as mean ± standard deviation or median (25% to 75%) interquartile range according to their distribution. Categorical variables are presented as counts and percentages. Comparison between the individual groups and comparison between BES and EES and BVS are performed on demographical data and risk factors as well as on procedural characteristics. The primary outcome is analyzed using a parametric or nonparametric test according to distribution and by the computation of a multivariate linear analysis by taking into account significant risk factors that potentially influence outcome thus adjusting for them. Clinical endpoints are compared using the Kaplan-Meier method. Binary logistic regression is equally performed in the search of independent predictors for the secondary endpoints. All analyses are performed according to the intention-to-treat principle. An intermediate analysis will be performed after follow-up angiography of 40 patients in each group.

### Trial interruption

The trial will be halted should the incidence of clinical endpoints significantly differ between the different devices during the intermediate analysis. The trial will also be halted should the incidence of adverse clinical events exceed the expected values at any point. The incidence of clinical events is under constant observation of the DSMB and CAEC. The principal investigator is continuously informed by the DSMB and CAEC and is, by not being blinded, solely responsible for trial halting.

## Discussion

Although DES have revolutionized percutaneous coronary interventions by significantly reducing clinically relevant restenosis and ‘target-lesion’ revascularization [17-20], numerous reports have since demonstrated an increased incidence of late stent thrombosis (LST) [21-23]. This is primarily due to a hypersensitivity reaction with secondary delayed arterial healing [24,25]. Substantial efforts have been made during the last decade to develop bioresorbable materials such as transient polymers or temporary ‘scaffolds’. The rationale for creating such devices was to lower both the neointimal proliferation in the short term and the hypersensitivity reaction (leading to late stent thrombosis) in the long term. Ideally, the device would offer an acute transient radial strength and later be completely absorbed thereby restoring vascular physiology.

Several DES using bioresorbable materials are currently available. The most used and studied so far is the Biomatrix™ BES. BES is a metallic stent that uses an abluminal bioresorbable polymer coating. This polymer is completely converted to lactic acid by 6 months and, via the Krebs cycle, to carbon dioxide and water by 6 to 9 months. More recently, a fully bioresorbable coronary scaffold has been launched for clinical use. The Absorb™ BVS is the first, fully bioresorbable coronary scaffold to have been launched for clinical use with CE approval. It uses a poly-l-lactide polymer that undergoes a four-stage bioresorption through hydration, depolymerization, polymer fragmentation and dissolution over 2 years via the Krebs cycle [26]. BVS was chiefly used in simple patients and lesions during early phases trials (ABSORB cohort A and B; ABSORB EXTEND). A second-generation device was developed to overcome the initial limitations of early scaffold shrinkage and provide greater radial support. Putative advantages over conventional DES are early restoration of physiological processes, namely vasomotion and remodeling, superior conformability, beneficial edge-vascular response and suppression of late-stent malapposition [27]. BVS absorption and vascular remodeling seem to seal stable plaques in settings of stable angina and type A lesions. With its widespread clinical launch in 2012, little is known on the broader use in more complex cases or acute coronary syndromes. The use of the BVS in complex situations makes sense however, as the risk of long-term complications from conventional DES implantation is even higher (longer lesions, chronic total occlusion, acute coronary syndromes and so on). For the time being, evidence has only provided information on its non-inferiority to modern DES in very specific settings. The EVERBIO II trial will assess BVS efficacy compared to the latest DES on the market in all patients viable for inclusion experiencing more complex lesions and clinical situations.

## Trial status

The recruitment phase is complete as of November 2013.

## Competing interests

SC has received educational grants from Abbott Vascular, Biosensors International and Boston Scientific. The other authors have no competing interests.

## Authors’ contributions

DA drafted this manuscript. SP performed the statistical planning. SJ, HV, EB and NC participated in data acquisition. MT, GB, JCS and JJG acquired the data and made critical revision of the manuscript for important intellectual content. SC conceived and designed the research, made critical revision of the manuscript for important intellectual content and handled funding and supervision. The authors read and approved the final manuscript.
